# TRIP-Br1 oncoprotein inhibits autophagy, apoptosis, and necroptosis under nutrient/serum-deprived condition

**DOI:** 10.18632/oncotarget.5072

**Published:** 2015-08-21

**Authors:** Samil Jung, Chengping Li, Jingjing Duan, Soonduck Lee, Kyeri Kim, Yeonji Park, Young Yang, Keun-Il Kim, Jong-Seok Lim, Chung-Il Cheon, Young-Sook Kang, Myeong-Sok Lee

**Affiliations:** ^1^ Department of Life Systems, Sookmyung Women's University, Seoul, 140-742, South Korea; ^2^ College of Pharmacy, Sookmyung Women's University, Seoul, 140-742, South Korea

**Keywords:** TRIP-Br1, autophagy, apoptosis, necroptosis, nutrient/serum starvation

## Abstract

TRIP-Br1 oncogenic protein has been shown to have multiple biological functions in cells. In this study, we demonstrate that TRIP-Br1 functions as an oncoprotein by inhibiting autophagy, apoptosis, and necroptosis of cancer cells and eventually helping them to survive under the nutrient/serum starved condition. TRIP-Br1 expression level was significantly increased in conditions with low levels of nutrients. Nutrient depleted conditions were induced by culturing cancer cells until they were overcrowded with high cell density or in media deprived of glucose, amino acids, or serum. Among them, serum starvation significantly enhanced the expression of TRIP-Br1 only in all tested breast cancer cell lines (MCF7, MDA-MB-231, T47D, MDA-MB-435, Hs578D, BT549, and MDA-MB-435) but not in the three normal cell lines (MCF10A, HfCH8, and NIH3T3). As compared with the control cells, the introduction of TRIP-Br1 silencing siRNA into MCF7 and MDA-MB-231 cells accelerated cell death by inducing apoptosis and necroptosis. In this process, TRIP-Br1 confers resistance to serum starvation-induced cell deaths by stabilizing the XIAP protein and inhibiting cellular ROS production. Moreover, our data also show that the intracellular increase of TRIP-Br1 protein resulting from serum starvation seems to occur in part through the blockage of PI3K/AKT signaling pathway.

## INTRODUCTION

Deregulation of cell death causes many human malignancies including cancers, in which cancer cells promote tumorigenesis by increasing resistance to cell death. For a long time, cell death was divided into two types: programed cell death (apoptosis) and non-programed cell death (necrosis). However, current concepts and techniques in cell death research have been changed dramatically. Eukaryotic cells are now thought to undergo different types of programed cell deaths (PCDs) through multiple signaling pathways. PCDs are now mainly divided into caspase-dependent or caspase-independent cell deaths [1–4]. Caspase-dependent PCD is a well-known apoptosis that proceeds via either the death receptor-mediated *extrinsic pathway* or the mitochondria-mediated *intrinsic pathway*. Caspase-independent PCD is a necroptosis, an alternative form of programed necrotic cell death mediated by receptor-interacting protein 1 and 3 (RIP1 and RIP3) kinases. Necrosis has long been considered to be a non-programed cell death. However, emerging evidences suggest that necrosis is also a kind of PCD and therefore, a new type PCD was proposed as the name of necroptosis by Xin Teng [5].

Cell death can be caused by many different types of stresses, one of which is extreme nutrient deficiency. Eukaryotic cells have evolved various cellular processes in response to nutrient starvation. At first, nutrient starvation causes autophagy, which contributes to cell survival by supplying additional nutrient and energy to starved cells [6–11]. When starvation is prolonged, sustained cellular digestion through repeated autophagic cycling eventually causes autophagy-dependent cell death [12–15]. Further extreme nutrient deficiency due to prolonged starvation eventually induces apoptosis and/or necroptosis [10, 11, 16, 17]. Unlike normal cells, cancer cells require unusually greater amounts of nutrients to support their enhanced growth and proliferation. In particular, highly aggressive and rapidly growing tumors are exposed to more severe nutrient deficiency. Accordingly, cancer cells are often vulnerable to nutrient stress and much more prone to cell death due to nutrient starvation when compared with normal cells. However, many cancer cells have developed a tolerance for nutrient depletion during tumorigenesis [18–20].

For many years, much effort has been focused on characterizing apoptosis in cancer research, and most of the currently available anticancer drugs have therefore been designed to kill tumor cells by triggering apoptosis. However, many malignant cancer cells have acquired strong resistant to those anticancer drugs owing to defects in their apoptotic PCD machinery (e.g., p53 mutations or defects of caspases). One of strategies to overcome this resistance is to exploit different types of cell deaths other than apoptosis. Many recent studies suggest that autophagy, apoptosis, and necroptosis pathways are connected to one another as mechanisms of cell death. Considering all these facts, finding ways to eliminate cancer cell's resistance to cell death may serve as an invaluable new strategy for the development of new anticancer drugs.

It is now widely accepted that most oncogenic proteins accelerate tumorigenesis by inhibiting autophagy, apoptosis, and necroptosis and therefore providing the resistance to cell death. In an effort to identify the proteins responsible for the resistance of cancer cells to nutrient starvation-derived cell death, we initially focused on the TRIP-Br1 (transcriptional regulator interacting with the PHD-bromodomain 1, also known as SERTAD1/SEI-1/p34^SEI-1^) oncoprotein. It has attracted increasing attention because of its various important biological functions, such as the regulation of cell-cycle progression, inhibition of apoptosis, and control of transcription, as well as a critical role in tumorigenesis [23–28]. However, little is known about its function under conditions of nutrient starvation. Our previous and current studies show that TRIP-Br1 has an anti-apoptotic characteristic and its expression was significantly increased in response to nutrient/serum starvation [23, 28]. Here, we report how TRIP-Br1 contributes to the survival of cancer cells in low-nutrient conditions during tumor growth.

## RESULTS

### Enhanced expression level of TRIP-Br1 under the condition of nutrient/serum starvation

**Figure 1:**
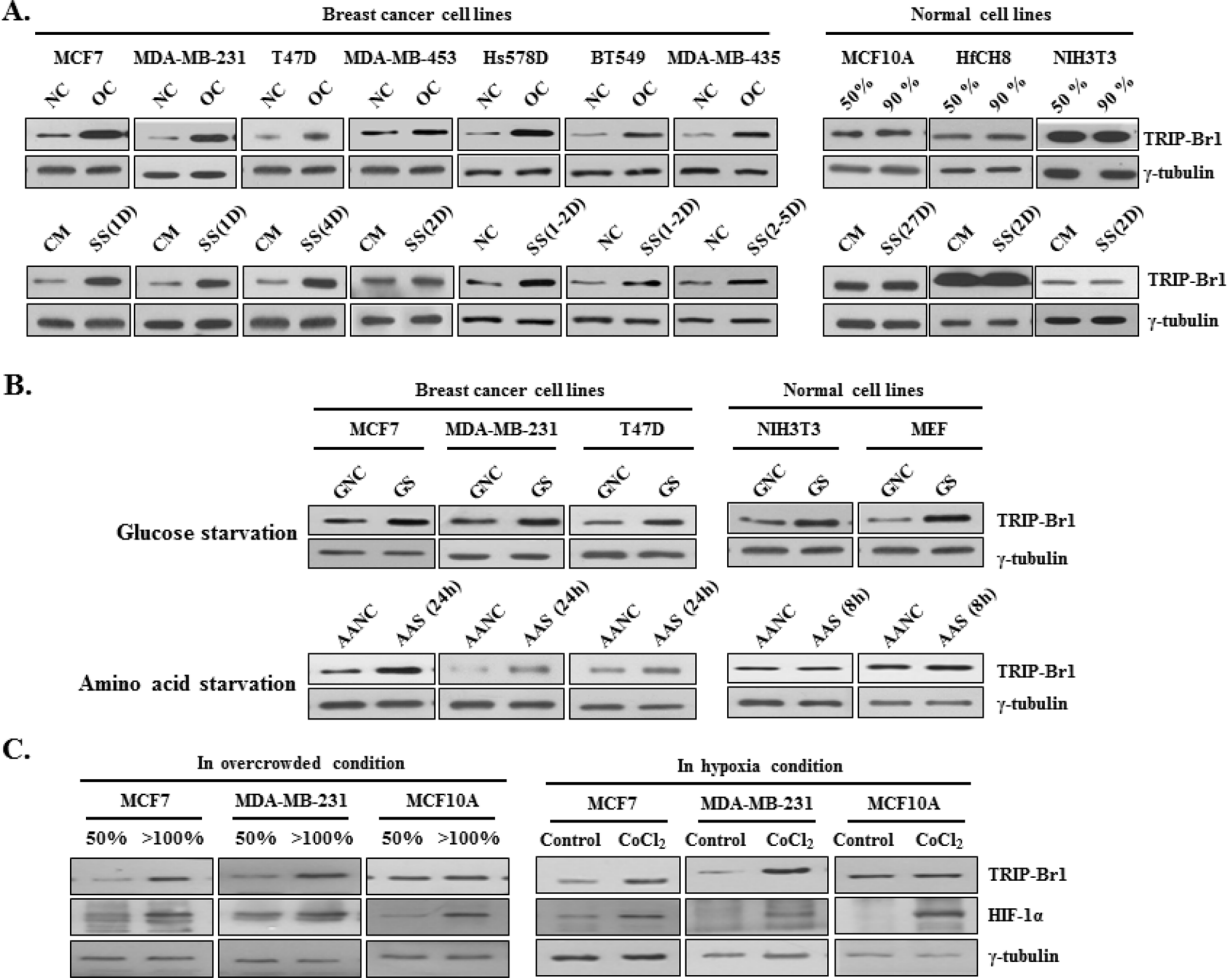
TRIP-Br1 expression levels in cancer and normal cells cultured under different conditions (media with or without serum, glucose, amino acids, or oxygen) **A.** Seven breast cancer and three normal cell lines were cultured in complete cell culture media until they either reached approximately 50% confluence (NC) or were overcrowded (OC), with high levels of cell confluence by prolonged culturing. Each cell line was also grown in a serum-containing complete medium (CM) or in a serum-starved medium (SS). Levels of TRIP-Br1 expression were checked by means of Western blot analysis after the indicated days (D) **B.** Both the cancer and normal cell lines were grown in a normal, complete DMEM medium containing sufficient glucose (GNC) or in a glucose-starved medium (GS) for 48 hours. Cells were also grown in an amino acid containing normal medium (AANC) or an amino acid-starved EBSS minimal medium (AAS). The cells were then collected, and TRIP-Br1 expression levels were measured by means of Western blot analysis. **C.** Both cancer and normal cell lines were cultured under either normoxic or hypoxic conditions; in the latter, hypoxia was induced by adding 500 μM of CoCl_2_ to the medium for 24 hours. The cells were then collected, and TRIP-Br1 expression levels were analyzed by using Western blot.

Both cancer and normal cells were cultured in normal complete (i.e., not starved) media until their cell density reached approximately 50% confluence or, with longer culturing, they became overcrowded with a high cell density. In the overcrowded condition, TRIP-Br1 expression was greatly increased in all seven of the breast cancer cell lines tested (MCF7, MDA-MB-231, T47D, MDA-MB-453, Hs578D, BT549, and MDA-MB-435) (Figure 1A). Interestingly, TRIP-Br1 expression did not increase in the normal human and mouse cell lines (MCF10A, HfCH8, and NIH3T3) probably because of their controlled growth and proliferation, prevented overcrowding (∼90% confluence and mono-layered). Next, we sought to determine what kind of stress might cause TRIP-Br1 up-regulation in cancer cells under the overcrowded condition. We found that TRIP-Br1 expression was not increased even at high levels of cell confluence when the media were exchanged for fresh media every 24 hours. Moreover, the increased TRIP-Br1 returned to normal levels within 24 hours after fresh media were added to the overcrowded cells (Supplementary Figure 1). It was therefore hypothesized that TRIP-Br1 up-regulation might be due to nutrient depletion resulting from the uncontrolled growth and proliferation of the cancer cells. To determine what kind of nutrient deficiency might be responsible for the increased TRIP-Br1 expression, selected cells were placed in conditions devoid of one of the three representative nutrients: serum, glucose, and amino acids. First, the effect of serum deficiency (the main form of stress used in this study) on TRIP-Br1 expression was examined in both cancer and normal cell lines. Serum is an important nutrient that is commonly used to support cell growth. Each cell line was grown in serum-containing complete medium or serum-starved medium. Interestingly, TRIP-Br1 gene expression was significantly increased in all the cancer cell lines tested but not in the normal cell lines, as was the case in the overcrowded condition (Figure 1A). The effect of glucose deficiency on TRIP-Br1 expression was also studied because glucose is the main source of nutrition and energy in most organisms. The selected cell lines were grown for 48 hours in media with either high or low levels of glucose. TRIP-Br1 expression increased within 48 hours in all the cancer cells and normal cells tested (Figure 1B). This result differed from the test involving serum starvation, indicating that the same mechanism might be at work in the regulation of TRIP-Br1 expression in both cell types. In a third experiment, the effect of amino acid deficiency on TRIP-Br1 expression was tested with the use of sufficient amino acid containing normal medium versus amino acid starved EBSS minimal medium. In the amino acid free medium, TRIP-Br1 expression increased within 24 hours in the cancer cells, whereas no significant change was detected in the normal cells (Figure 1B). However, it may not be appropriate to compare TRIP-Br1 protein levels in this case with those of other cancer cells because TRIP-Br1 expression was measured much earlier than in the other two tests of nutrient stress. In normal cells, TRIP-Br1 expression level was measured at 8 hours after amino acid starvation because the cells began to die relatively early (Figure 1B). This indicates that the normal cells are much more vulnerable to amino acid deficiency than the cancer cells. Considering all these data, we chose the serum starved condition for further study because TRIP-Br1 expression was increased only in the cancer cells, as was the case for the overcrowded condition.

In an extension of this study, the effect of hypoxic or acidic conditions on TRIP-Br1 expression was also tested because they are also characteristics of cancer cells in an overcrowded condition. The condition of hypoxia was induced by adding 500 μM of CoCl_2_ to the cells for 24 hours. TRIP-Br1 expression was increased in the MCF7 and MDA-MB-231 cancer cells but not in the MCF10A normal cells (Figure 1C). This result indicates that TRIP-Br1 expression was also increased at least in part by hypoxia caused by overcrowd condition. Leontieva OV *et al* suggested that loss of viability of overgrown “yellow” cancer cell culture is caused by the acidification of media (pH 6.8) due to lactate overproduction [30]. Because acidification of media due to lactate production is also one of causes of overgrown cell death, it was suspected that overgrown cell-derived acidification of media might be responsible for the increased TRIP-Br1 expression level. Therefore, it was tested in acidic media, which was prepared by adding HCl to growth media (pH 6.8 and 7.0). However, TRIP-Br1 expression was not increased in the acidic media (data not shown), suggesting that overgrown cell-mediated acidic condition has no effect on TRIP-Br1 expression level.

In conclusion, our data show that TRIP-Br1 gene expression was significantly increased at the protein level by nutrient/serum deficiency in all the tested breast cancer cells but not in the normal cells.

### Negative effect of TRIP-Br1 on serum starvation-induced cell death

**Figure 2:**
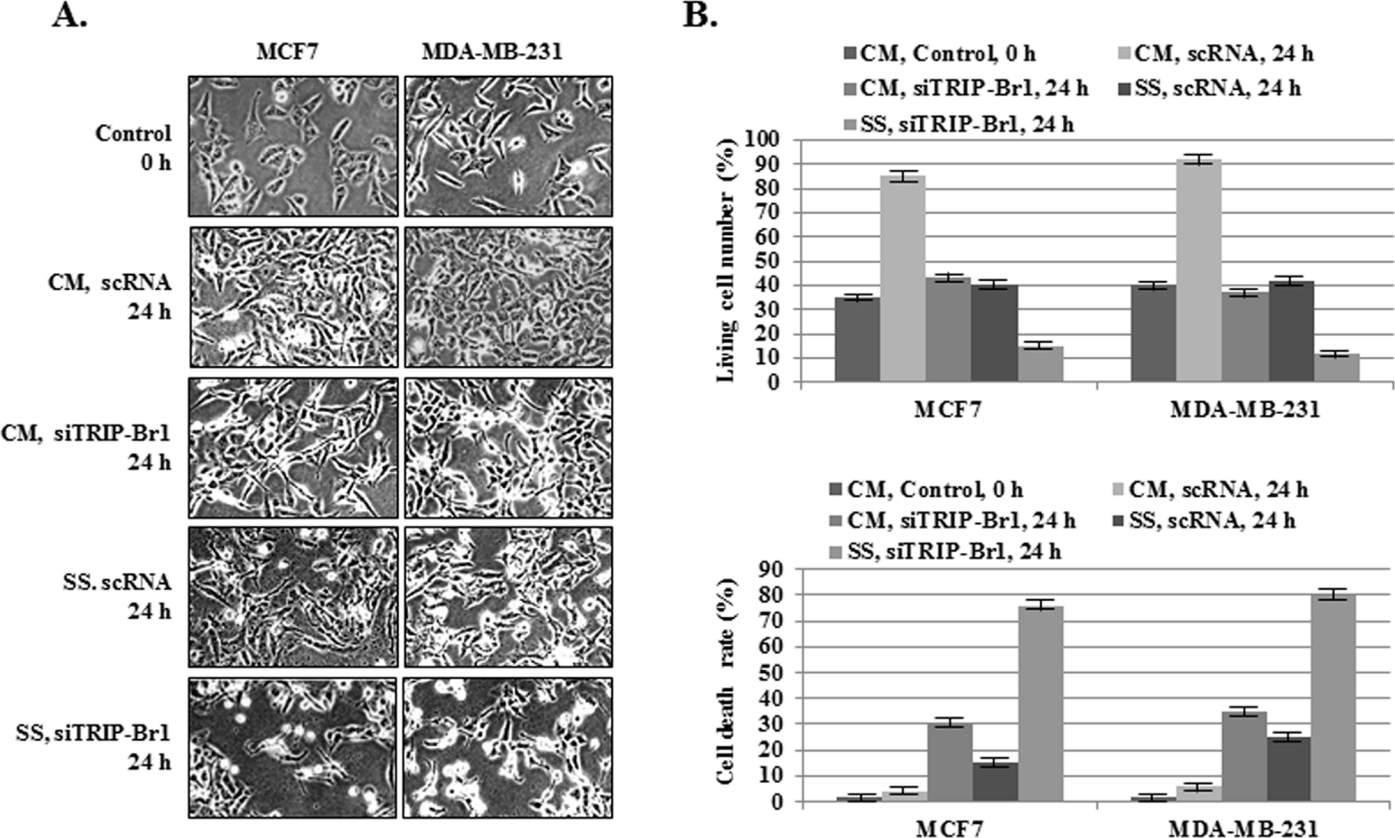
Inhibitory role of TRIP-Br1 in serum starvation-induced cell death MCF7 and MDA-MB-231 cells were transiently transfected with scrambled RNA (scRNA) or TRIP-Br1 silencing siRNA (siTRIP-Br1). The cells were then incubated in media with or without serum for 24 hours. **A.** After transfection with scRNA or siTRIP-Br1, the phenotypes of the MCF7 and MDA-MB-231 cells were photographed under the microscope in both normal and serum-starved conditions. **B.** The percentages of living and dead cells were evaluated by means of trypan blue-staining after transfection with scRNA or siTRIP-Br1 under normal and serum-starved conditions. The data represent the means ± SD from the three independent experiments. *P* < 0.05.

In spite of the fact that nutrient deficiency is more deleterious to cancer cells than to normal cells, many cancer cells overcome this stressful condition by controlling specific regulatory system or proteins. In this study, we showed that TRIP-Br1 gene expression was significantly increased after serum starvation only in breast cancer cells but not in normal cells. Our previous report also showed that TRIP-Br1 endows cancer cells with anti-apoptotic properties in response to anticancer drugs [23]. We therefore hypothesized that TRIP-Br1 up-regulation might contribute to the enhanced survival of cancer cells under conditions of nutrient/serum deficiency. This hypothesis can be supported by the finding that TRIP-Br1 silencing in MCF7 and MDA-MB-231 cells accelerated cell death when these cells, as compared with control cells, were put in serum-depleted media and even when they were in normal conditions (Figure 2A, and 2C). This data strongly suggest that TRIP-Br1 has a positive effect on cancer cell survival in conditions of nutrient/serum starvation.

Taken together, these observations strongly suggest that TRIP-Br1 confers resistance to nutrient/serum starvation-induced cell death in cancer cells.

### Inhibitory role of TRIP-Br1 in autophagy, apoptosis, and necroptosis in serum starved condition

It is well known that prolonged overcrowded and serum-depleted conditions ultimately induce cell death. Thus, we wished to determine what kinds of cell deaths can be induced by these conditions. MCF7, MDA-MB-231 breast cancer, and MCF10A normal cells were incubated in serum containing normal medium until the cells became overcrowded or in serum-free media for 24 or 48 hours, along with controls. The cells were then collected and subjected to the Western blot analysis. Three representative types of cell deaths (autophagy, apoptosis, and necroptosis) were studied by means of corresponding biomarkers or correlated regulatory proteins.

**Figure 3:**
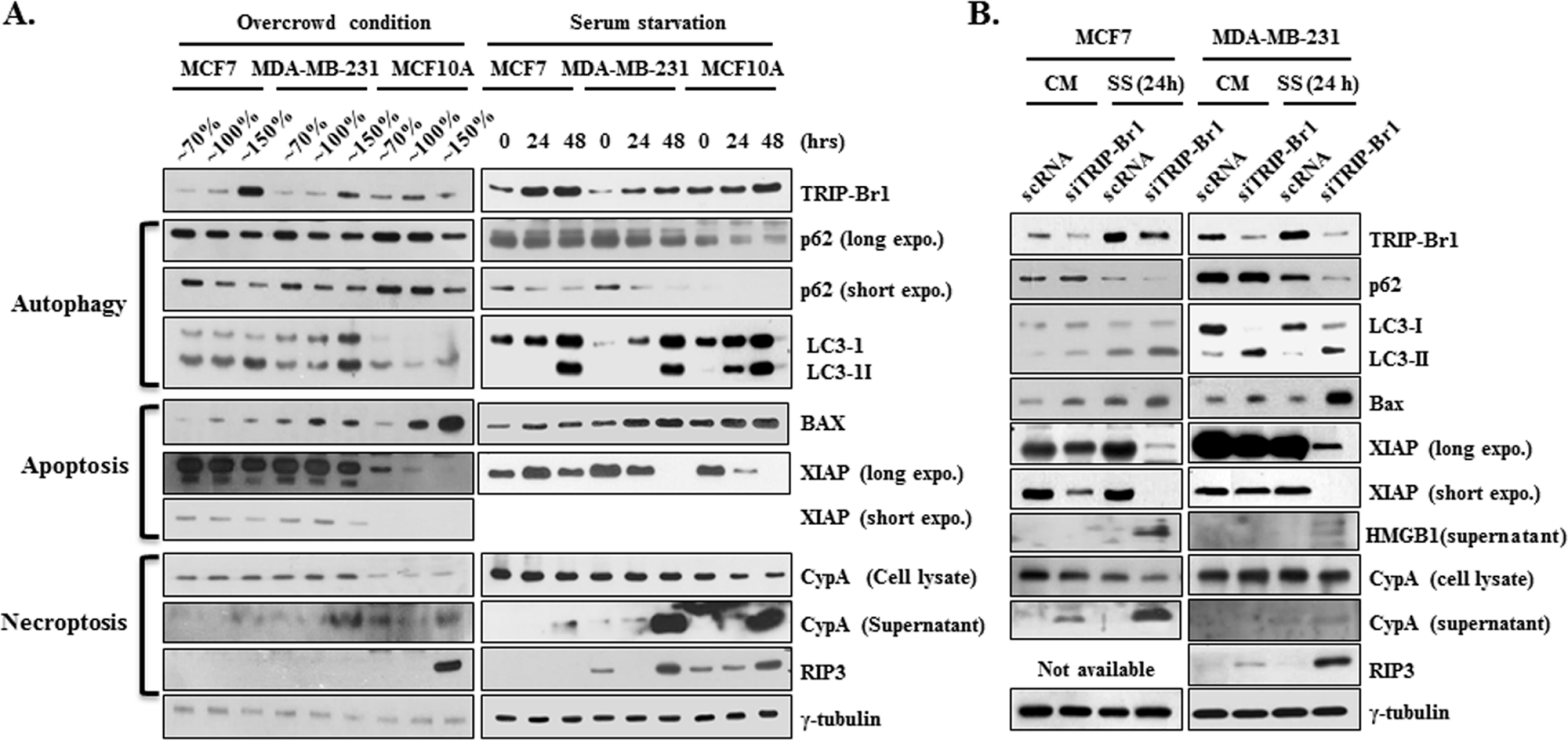
Inhibitory role of TRIP-Br1 in autophagy, apoptosis, and necroptosis **A.** Autophagy, apoptosis, and necroptosis were checked in MCF7 and MDA-MB-231 cancer and MCF10A normal cell lines under the conditions of overcrowding or serum starvation. The cells were grown until either they became overcrowded with high cell densities or they were maintained in DMEM or DMEM/F12 media that did not contain FBS or horse serum for 24 or 48 hours. After the time periods indicated had elapsed, the cells were collected and subjected to Western blot analyses. γ-tubulin was used as a control marker. **B.** MCF7 and MDA-MB-231 cells were transfected with scrambled RNA (scRNA) or TRIP-Br1 silencing siRNA (siTRIP-Br1) and then incubated in media with or without serum for 24 or 48 hours. Western blot analysis was employed to determine the effect of TRIP-Br1 on autophagy, apoptosis, and necroptosis by means of corresponding biomarkers and correlated regulatory proteins. CM: normal condition with complete media; SS: serum-starved condition for 24 or 48 hours. The loading control was γ-tubulin.

At first, autophagy was assessed using two well-known autophagic markers, p62 and LC3. As expected, both conditions stimulated autophagy. The p62 expression level was decreased and the conversion ratio from LC3-I to LC3-II was enhanced in response to both stresses (Figure 3A). Next, the effect of both conditions on apoptosis was assessed by measuring the levels of expression of apoptotic marker protein (Bax) or of regulatory protein (XIAP). Both stressful conditions resulted in significant increase in the Bax level (Figure 3A). In our earlier study, we found that TRIP-Br1 inhibits the apoptosis of cancer cells by stabilizing XIAP, a potent apoptosis inhibitor [23]. Therefore, we tested XIAP stability, in which XIAP level was found to be decreased, indicating that these stressful environments indeed induce apoptosis. Finally, necroptosis was examined with the use of the necroptosis marker CypA under same conditions [29]. The export of CypA protein into the extra-cellular environment was increased in all three cell lines (Figure 3A), indicating the induction of necroptosis. Expression of RIP3, one of the essential genes for necroptosis, was increased in the MDA-MB-231 and MCF10A cells, whereas RIP3 expression was not detected in the MCF7 cells because of its absence (Figure 3A). Altogether, these results strongly suggest that both overcrowded and serum-depleted conditions can trigger autophagy, apoptosis, and necroptosis in both cancer cells and normal cells. However, MCF10A normal cells seem to be more sensitive to both stressful conditions compared with MCF7 and MDA-MB-231 cancer cells.

To determine whether TRIP-Br1 is involved in autophagy, apoptosis, and necroptosis under these stressful environments, MCF7 and MDA-MB-231 cells were transiently transfected with scrambled RNA (scRNA) or TRIP-Br1 silencing siRNA (siTRIP-Br1) and then incubated in media with or without serum for 24 hours. Interestingly, our Western blot analysis revealed that TRIP-Br1 is responsible for all of autophagy, apoptosis, and necroptosis (Figure 3B). It is now widely accepted that most oncoproteins inactivate autophagy whereas tumor suppressors mostly activate it [21, 22]. Considering that TRIP-Br1 is an oncoprotein, it was thought that TRIP-Br1 might inhibit autophagy, and our data confirmed this hypothesis. The introduction of TRIP-Br1 silencing siRNA into MCF7 and MDA-MB-231 cells led to more rapid processing of the p62 down-regulation and the conversion of LC3-I to LC3-II, as compared with control cells in serum-free media and even in complete media (Figure 3B). This result strongly suggests that TRIP-Br1 inhibits autophagy. Further study showed that the level of expression of Beclin 1, an important protein in autophagy, did not respond to different levels of TRIP-Br1 in the MCF7 and MDA-MB-231 cells (data not shown). On the contrary, the effect of autophagy on TRIP-Br1 gene expression was also tested by using monensin, a late-stage autophagy inhibitors, which did not change TRIP-Br1 expression in either the normal or the serum-starved condition (data not shown). Altogether, our data strongly suggest that TRIP-Br1 inhibits autophagy under the condition of serum starvation, but the inhibition of autophagy itself has no effect on TRIP-Br1 expression in either condition. Next, we tested the effect of TRIP-Br1 on apoptosis, in which a slight up-regulation of Bax was detected in the TRIP-Br1 silenced MCF7 and MDA-MB-231 cells (Figure 3B). Our data also show that the expression levels of TRIP-Br1 and XIAP are coordinated in MCF7 and MDA-MB-231 cancer cells. XIAP protein was greatly decreased when TRIP-Br1 expression was silenced, particularly during serum starvation (Figure 3B). This result suggests that TRIP-Br1 protein inhibits apoptosis at least in part by stabilizing XIAP under the stressful condition of serum starvation. Finally, the effect of TRIP-Br1 on necroptosis was examined by means of two useful markers, HMGB1 and CypA [29]. The export of HMGB1 and CypA proteins into the extra-cellular environment was slightly increased in TRIP-Br1 silenced cells during serum starvation (Figure 3B). In addition, immunoreactive RIP3 was found to be greatly increased in TRIP-Br1 silenced MDA-MB-231 cells during serum depletion (Figure 3B), suggesting that TRIP-Br1 has an inhibitory role in necroptosis induced by serum starvation.

In conclusion, our results strongly suggest that both overcrowded and serum-depleted conditions induce the autophagy, apoptosis, and necroptosis, and TRIP-Br1 render cancer cells more resistant to such cell deaths.

### Inhibitory role of TRIP-Br1 in autophagy, apoptosis, and necroptosis through its positive effect on XIAP

**Figure 4:**
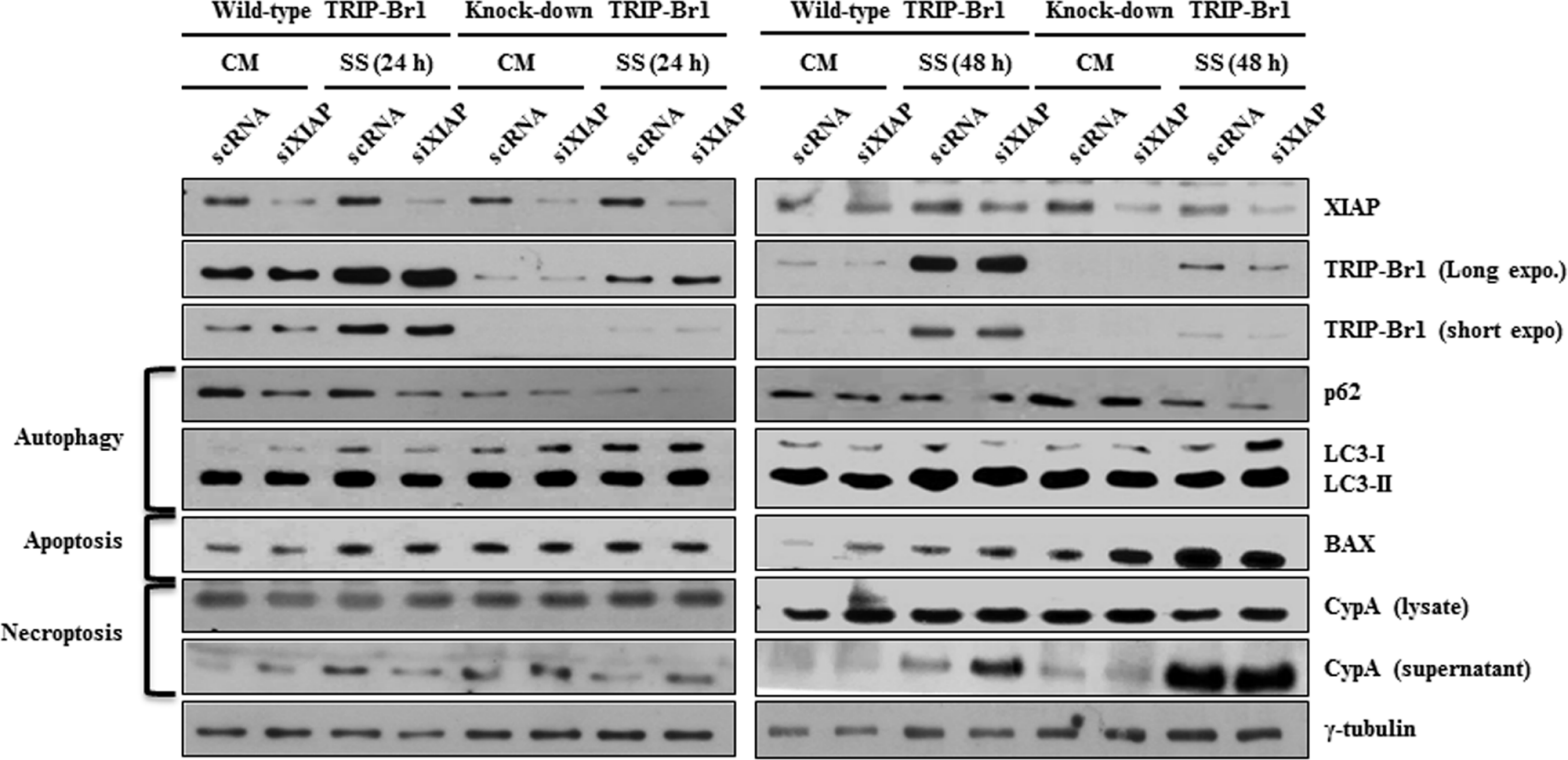
The negative effect of TRIP-Br1 and/or XIAP on autophagy, apoptosis, and necroptosis under the condition of serum starvation TRIP-Br1 wild-type or knock-down MCF7 stable cell line was transiently transfected with XIAP silencing siRNA (siXIAP) (see Materials and Methods), in which scrambled RNA (scRNA) was used as a nonsilencing control. Cells were serum-starved for 24 or 48 hours, and the effect of TRIP-Br1 and/or XIAP on autophagy, apoptosis, and necroptosis was tested by employing Western blotting with each marker or regulatory proteins.

The present study showed that increased TRIP-Br1 under the nutrient/serum-starved condition is at least in part responsible for the inhibition of the autophagy, apoptosis, and necroptosis in cancer cells. Accordingly, we then wished to determine the mechanism underlying this effect. At first, we focused on the effect of TRIP-Br1 on XIAP expression in serum starvation. Our previous report showed that TRIP-Br1 inhibits cancer cell death by stabilizing XIAP through direct binding with it [23]. XIAP is a member of the inhibitor of apoptosis family of proteins (IAP) and the most potent human IAP currently identified. It can effectively prevent cell death induced by various stimuli such as tumor necrosis factor alpha (TNF-α), Fas, ultraviolet, and genotoxic agents [31]. It is well known that XIAP stops apoptotic cell death by binding to and inhibiting caspases 3, 7, and 9 [32–38]. In addition, Huang *et al*. suggested that XIAP also inhibits autophagy by suppressing XIAP-Mdm2-p53 signaling [39]. Considering the strong relationship between TRIP-Br1 and XIAP and the inhibitory role of XIAP in apoptosis and autophagy, it was hypothesized that XIAP also might inhibit necroptosis in serum starvation. To test this hypothesis, XIAP silencing siXIAP was introduced into TRIP-Br1 wild-type and knock-down MCF7 cells, and its effect was tested with each corresponding marker. As shown in Figure 4, XIAP inhibited necroptosis as well as apoptosis and autophagy, which was interestingly maximized by TRIP-Br1. Our data show that autophagy, apoptosis, and necroptosis were accelerated in MCF7 cells with the double mutants of XIPA and TRIP-Br1, suggesting that TRIP-Br1 inhibits them at least in part by working with XIAP.

### Inhibition of autophagy, apoptosis, and necroptosis by TRIP-Br1 through its repression of ROS production in serum-starved condition

**Figure 5:**
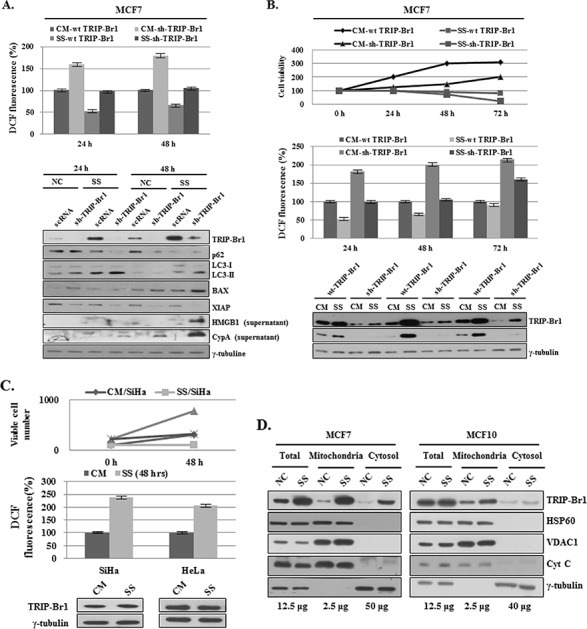
Inhibition of autophagy, apoptosis, and necroptosis by TRIP-Br1 through its repression of ROS production in serum-starved condition **A.** MCF7 cells were cultured in complete or serum-starved media for 24 and 48 hours. After the cells were collected and 2.5 × 10^4^ MCF7 cells were plated in 96 wells, ROS levels were measured after adding DCFH-DA (see Materials and Methods). The data represent the means ± SD from the three independent experiments. *p* < 0.05. The cells were then used for the analysis of autophagy, apoptosis, and necroptosis by means of Western blotting. **B.** Cells were grown in a serum-containing complete medium (CM) or in a serum-starved medium (SS) for 24, 48, and 72 hours. The percentage of viable cells was evaluated by means of trypan blue-staining. ROS levels were measured as described in the section of Materials and Methods. The data represent the means ± SD from the three independent experiments. *p* < 0.05. The induction of autophagy, apoptosis, and necroptosis was analyzed by means of Western blot analysis. **C.** ROS levels in SiHa and HeLa cervical cancer cell lines. Cells were grown in complete (CM) or serum-starved media (SS) for 24 or 48 hours. The percentages of living and dead cells were evaluated by means of trypan blue-staining. ROS levels were measured as described in the section of Materials and Methods. The data represent the means ± SD from the three independent experiments. *p* < 0.05. The TRIP-Br1 protein level was checked by using Western blotting. **D.** Mitochondrial TRIP-Br1 was purified from MCF7 cancer and MCF10A normal cells after these cells were placed in normal and serum-starved conditions. Mitochondrial fractionation was performed as previously described [23], in which each marker protein was used as following; HSP60 for nuclear marker, VDAC1 and CytC for mitochondrial marker, and γ-tubulin for cytosolic fractionation marker.

To elucidate the inhibitory role of TRIP-Br1 in the three types of cell deaths induced by nutrient/serum starvation, we also tested the effect of TRIP-Br1 on intracellular ROS level. At physiologic levels, ROS play vital roles in cellular metabolism, cell signaling, and homeostasis as main cellular regulators. However, ROS levels greatly increase in stressful environments (e.g., ultraviolet, heat exposure, or nutrient stress), eventually resulting in significant damage to cell structures and ultimately cell death [40]. ROS can induce autophagy, apoptosis, and necroptosis depending on their cellular levels [40]. Somewhat higher levels of ROS can induce cell death through autophagy [15]. Excessive ROS level induces apoptosis through both the extrinsic and intrinsic pathways, and an even higher ROS level causes necroptosis [41–45]. Because cancer cells are known to produce higher levels of ROS than normal cells do, one might ask how cancer cells survive with much higher ROS levels in a stressful environment such as nutrient starvation. This survival might be explained in part by another important cellular function of TRIP-Br1 in cancer cells. Our previous study showed that TRIP-Br1 inhibits ROS-induced cell death by suppressing ASK1 (apoptosis signal-regulating kinase 1), a key factor in the control of several cellular events, including the cell cycle, senescence, and apoptosis [27]. Thus, it is plausible to assume that TRIP-Br1 may inhibit the other two types of PCDs by negatively regulating ROS generation. To test this hypothesis, a TRIP-Br1 silenced stable cell line was constructed (see Materials and Methods). Resulting MCF7 cells with wild-type or knock-down TRIP-Br1 were grown in complete or serum starved media for 24 or 48 hours, and intracellular ROS content was evaluated by means of 2′,7′-dichlorodihydrofluorescein. In TRIP-Br1 silenced cells, ROS levels were about 1.6 and 1.9 times higher than those in TRIP-Br1 wild-type cells in complete and serum starved media, respectively (Figure 5A). Interestingly, serum starvation seems to induce different types of cell death depending on how long the cells are exposed to serum starvation. At first, serum deficiency induced autophagy at 24 hours, but prolonged serum starvation triggered the other two types of cell deaths, apoptosis and necroptosis (Figure 5A). To clarify the effect of TRIP-Br1 on PCDs, both stable cell lines were grown in complete or serum starved media for 24, 48, or 72 hours, and growth curves, ROS levels, and TRIP-Br1 expression levels were all checked at the same time (Figure 5B). Growth was found to be much faster in the TRIP-Br1 wild-type cells than in the TRIP-Br1 silenced cells (Figure 5B). ROS levels started to increase in proportion to the length of exposure to serum starvation in both TRIP-Br1 wild-type and knock-down cells (Figure 5B). Importantly, an inverse relationship was observed between the levels of ROS and TRIP-Br1 expression, suggesting the inhibitory role of TRIP-Br1 in ROS production (Figure 5B). It is generally accepted that serum deprivation stimulates ROS production. Our results showed that ROS levels in MCF7 cells cultured in serum-free medium were at first lower than those in MCF7 cells grown in complete media at 24 hours. However, after 48 hours, they eventually start to increase. Lower ROS levels in early times might be due to increased TRIP-Br1 in response to serum depletion. To test this assumption, the SiHa and HeLa cervical cell lines were chosen, in which TRIP-Br1 expression was not increased in serum-depleted conditions. In this case, ROS levels were significantly increased in these cell lines in response to serum starvation (Figure 5C). Then, how can TRIP-Br1 inhibit intracellular ROS production? It is well known that ROS are generated mainly inside mitochondria. We previously suggested that TRIP-Br1 protein level was significantly increased in the nucleus and cytosol in response to serum starvation [28]. In this study, TRIP-Br1 protein level in mitochondria was also checked under the condition of serum starvation. Interestingly, present data show that TRIP-Br1 expression was significantly increased in the mitochondria of MCF7 but not in the mitochondria of MCF10A cells (Figure 5D). Mitochondrial metabolism usually produces physiologic ROS to support cell survival. However, mitochondria that have been damaged as a result of various stresses produce much higher levels of ROS and ultimately induce all three autophagy, apoptosis, and necroptosis depending on their different ROS levels [46]. Considering all these facts, it may be plausible to assume that increased TRIP-Br1 protein in the mitochondria inhibits ROS production and finally alleviates autophagy, apoptosis, and necroptosis.

In conclusion, TRIP-Br1 up-regulation in cancer cells inhibits autophagy, apoptosis, and necroptosis at least in part by repressing ROS production under the condition of serum starvation.

### Negative effect of TRIP-Br1 on the hydrogen peroxide (H_2_O_2_) induced cell death

**Figure 6:**
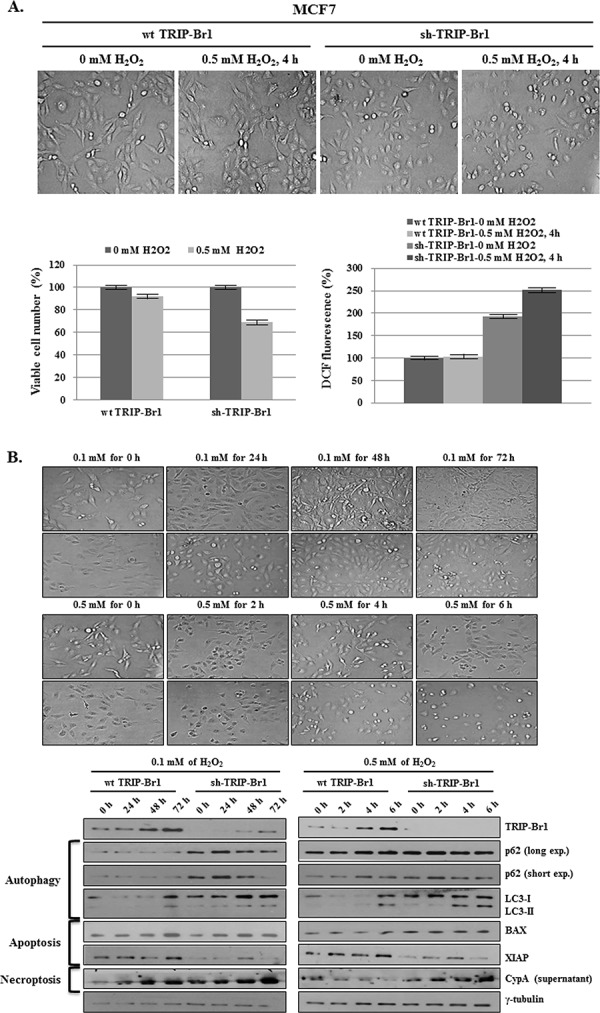
Inhibitory role of TRIP-Br1 in hydrogen peroxide (H_2_O_2_) induced cell death **A.** After TRIP-Br1 wild-type or knock-down MCF7 cells were treated with 0.5 mM of H_2_O_2_ for 4 hours, the microscopic phenotype was photographed, the number of viable cells was counted by means of trypan blue staining, and ROS levels were measured. The data represent the means ± SD from the three independent experiments. *p* < 0.05. **B.** Wild-type or knock-down TRIP-Br1 cells were treated with 0.1 or 0.5 mM of H_2_O_2_ for the indicated time periods, and Western blot analysis was employed to check the induction of autophagy, apoptosis, and necroptosis.

Our data suggest that increased TRIP-Br1 expression attenuated autophagy, apoptosis, and necroptosis by suppressing ROS production under the condition of serum starvation. To support this conclusion, we also tested the effect of TRIP-Br1 on H_2_O_2_ induced cell death. Current data show that overcrowding cell condition leads to a hypoxic condition as well as nutrient starvation. Vergara *et al*, suggested that a hypoxic condition induces H_2_O_2_ production [47]. To find out what condition could cause cell death, TRIP-Br1 wild-type or knock-down MCF7 cells were treated with increasing concentrations of H_2_O_2_ (0, 0.1, 0.5, and 1.0 mM) for 24 hours. In the cells treated with 0.1 mM of H_2_O_2_, slow cell growth was noted but no cell death was detected. Cells treated with 0.5 mM of H_2_O_2_ started to die at 8 hours. However, at its highest concentration (1.0 mM), H_2_O_2_ triggered cell death at very early time (Supplementary Figure 2). Therefore, 0.5 mM of H_2_O_2_ was chosen for the further study, and cells were treated for 4 hours. As expected, cell viability was significantly higher in TRIP-Br1 wild-type cells, as compared with TRIP-Br1 silenced cells (Figure 6A). Importantly, intracellular ROS levels were found to be much higher in TRIP-Br1 silenced cells in both complete and serum starved conditions (Figure 6A). In an extended study, the effect of TRIP-Br1 on H_2_O_2_-induced cell deaths was also tested. After MCF7 cells were treated with 0.1 or 0.5 mM of H_2_O_2_ for the time periods indicated, the cells were photographed, and corresponding marker proteins were used to detect the autophagy, apoptosis, and necroptosis. The results were similar to those seen under the condition of serum starvation. H_2_O_2_ treatment induced autophagy at a relatively early time (at 24 hours) and then apoptosis and necroptosis later on (at 48 and 72 hours) (Figure 6B). Importantly, TRIP-Br1 attenuates all the autophagy, apoptosis, and necroptosis in response to H_2_O_2_ treatment, as shown that all of them were clearly accelerated in TRIP-Br1 silenced MCF7 cells (Figure 6B).

Taken together, our result strongly suggests that TRIP-Br1 inhibits autophagy, apoptosis, and necroptosis in response to H_2_O_2_ treatment, as is the case of serum starvation-induced condition.

### Effect of PI3K/AKT signaling pathway on TRIP-Br1 expression under the condition of serum depletion

**Figure 7:**
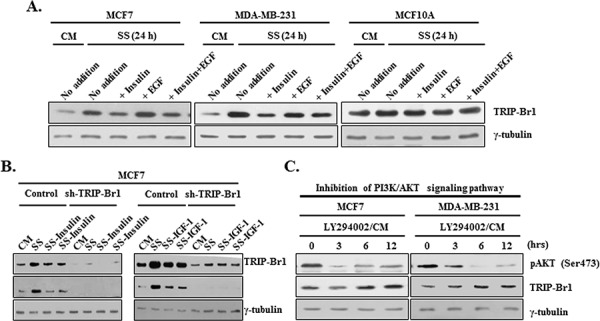
Effect of PI3K/AKT signaling pathway on TRIP-Br1 expression in serum-depleted condition **A.** Effect of insulin survival factor and EGF on TRIP-Br1 expression. MCF7, MDA-MB-231, and MCF10A cells were treated with 10 μg/ml of insulin or 50 μM of EGF for 24 hours in the presence or absence of serum. TRIP-Br1 gene expression was assessed by means of Western blot analysis. **B.** Cells were treated with 20 or 40 μg/ml of insulin and 300 or 400 ng/ml of IGF for 24 hours in the absence of serum. TRIP-Br1 gene expression was assessed by Western blot analysis. **C.** Cells were treated with 50 μM of LY294002, PI3K/AKT pathway inhibitor in the complete media with serum. The change in the level of TRIP-Br1 expression was checked using Western blot analysis. Dephosphorylation on the Ser473 residue of AKT was used as a marker for the inhibition of PI3K/AKT signaling pathway. Cells were grown in a serum-containing complete medium (CM) or in a serum-starved medium (SS).

We have shown that serum deficiency increased TRIP-Br1 expression only in cancer cells but not in normal cells, thereby providing cancer cells with enhanced resistance to the autophagy, apoptosis, and necroptosis. These facts imply that cancer cells sense a ‘serum-free’ environment differently or translate that environmental signal into survival signal. There should be a specific molecular mechanism by which cancer cells acquire the ability to survive under serum-depleted conditions. Accordingly, our next question was what mechanism is responsible for the up-regulation of TRIP-Br1 in cancer cells under the condition of serum starvation. It is well known that serum contains many different types of survival and growth factors including insulin and EGF, even though its exact and complete composition is still unknown. Insulin and EGF are well known potent survival and growth factors, respectively. We suspected that increased TRIP-Br1 expression might be due to a deficiency of survival or growth factors in serum-free media. Therefore, we tested the effects of insulin and EGF on TRIP-Br1 expression. Insulin and/or EGF were added to DMEM medium without FBS or horse serum. Interestingly, the effect of serum deficiency on TRIP-Br1 protein level was reversed by the addition of insulin but not by EGF (Figure 7A). However, TRIP-Br1 protein levels again started to increase after 48 hours, probably owing to the exhaustion of insulin (data not shown). Lin *et al*. suggested that the addition of EGF to media compensated for the effect of growth factor deficiency [7]. In our study, however, the levels of TRIP-Br1 expression did not respond to the addition of EGF (Figure 7A). Thus, TRIP-Br1 expression seems to be at least in part increased by insufficient insulin survival factor in MCF7 and MDA-MB-231 cancer cells under the condition of serum starvation. It is known that insulin-like growth factor 1 (IGF1) binds to the same receptor (e.g., IGF1R) and transmits subsequent signaling through the same PI3K/AKT signaling pathway. Thus, IGF1 was also added to serum-free media and similar result was obtained (Figure 7B). Our data suggest that insulin and IGF1 survival factors appear to have a major impact on TRIP-Br1 expression. They respond to a number of different apoptotic triggers, and this effect is stimulated through the PI3K/AKT signaling pathway [48, 49]. Ng *et al*. suggested that insufficient survival signals usually inactivate the PI3K signaling pathway and induce autophagy and apoptosis [50]. Considering all these facts, it was assumed that TRIP-Br1 expression might be increased by the inhibition of PI3K/AKT signaling due to the depletion of survival factors under the condition of serum starvation. Thus, we initially focused on the PI3K/AKT signaling pathway, a well-known important pathway for the proliferation and survival of cancer cells. The effect of the PI3K/AKT signaling pathway on TRIP-Br1 gene expression was examined using LY294002, a PI3K/AKT inhibitor, in normal or serum starved conditions. As shown in Figure 7C, inhibition of the PI3K/AKT signaling pathway significantly increased the TRIP-Br1 expression in MCF7 and MDA-MB-231 cells under normal conditions.

Taken together, these data suggest that blockage of the PI3K/AKT signaling pathway due to serum starvation is at least partly responsible for the increase of TRIP-Br1 gene expression in cancer cells.

## DISCUSSION

**Figure 8:**
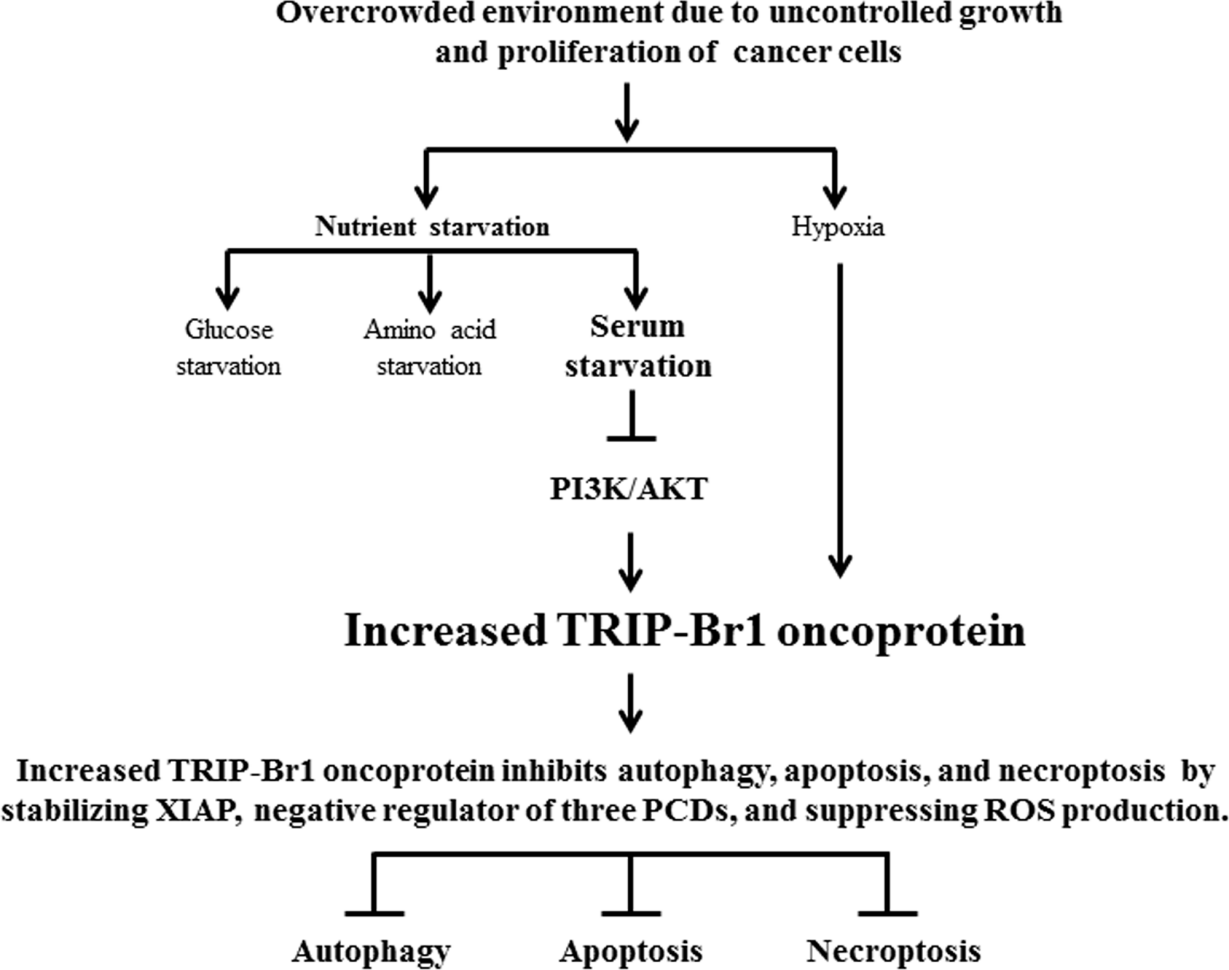
Schematic diagram showing the inhibitory role of TRIP-Br in autophagy, apoptosis, and necroptosis under nutrient/serum-deprived condition Overcrowding, hypoxia, and nutrient/serum-starved conditions caused the up-regulation of TRIP-Br1 in the cancer cells but not in the normal cells. Nutrient/serum starvation induces autophagy, apoptosis, and necroptosis to which increased TRIP-Br1 oncoprotein confers resistance in cancer cells. Increased TRIP-Br1 inhibits autophagy, apoptosis, and necroptosis in cancer cells in part by stabilizing XIAP and/or by inhibiting ROS production. Serum starvation causes the increase of TRIP-Br1 expression partly through the blockage of the PI3K/AKT signaling pathway.

Our ultimate goal is to establish an optimal strategy to selectively kill cancer cells and to find possible new biomedical candidates for cancer therapy. For this purpose, we initially tried to identify the extracellular stimuli and key elements that respond differently to cell death in normal and cancer cells. Nutrient deficiency is a common stressful condition that cancer cells encounter *in vivo* owing to their uncontrolled growth, indicating that nutrient deficiency is much more deleterious to cancer cells than to normal cells. The problem is that many cancer cells overcome this stressful environment by developing different mechanism(s) for survival. This fact implies that normal and cancer cells respond differently when faced with nutrient starvation, especially serum deficiency. In an effort to elucidate the mechanism responsible for this, our results suggest that serum deficiency enhanced the expression of TRIP-Br1 oncogenic protein only in cancer cells but not in normal cells. TRIP-Br1 helps cancer cells to survive by suppressing autophagy, apoptosis, and necroptosis. Our summary model shows the mechanism how TRIP-Br1 functions as an oncogenic protein in response to nutrient/serum starvation (Figure 8).

TRIP-Br1 was first identified as an antagonist of p16 that facilitates the formation and activation of cyclin D-Cdk4 complexes [24]. TRIP-Br1 directly binds to Cdk4 and inhibits the activities of p16 and finally Rb [51]. It is now known that most oncoproteins inactivate autophagy while tumor suppressors activate it [21, 22]. In fact, current data showed that up-regulation of the TRIP-Br1 oncoprotein inhibited autophagy in cancer cells. Thorburn *et al*. showed that p16 and retinoblastoma (Rb) tumor suppressors activate autophagy [52]. Concerning the inhibitory role of TRIP-Br1 in autophagy, it seems plausible to assume that TRIP-Br1 may also inhibit autophagy by negatively regulating the activity or stability of other tumor suppressors such as p16 and RB.

TRIP-Br1 seems to inhibit autophagy, apoptosis, and necroptosis by controlling related regulatory proteins or pathways in different sub-cellular locations. Interestingly, our previous and current studies revealed a significant increase of TRIP-Br1 protein in mitochondria as well as nucleus and cytosol upon serum starvation [28]. Mitochondria are the essential site of aerobic energy production in eukaryotic cells, and therefore damage to the mitochondria usually causes disease. Accordingly, the well-being of cells requires the maintenance of healthy mitochondria and the elimination of dysfunctional mitochondria. The major degradative pathway for turnover of damaged mitochondria is mitophagy, mitochondrial degradation by autophagy [53]. Rambold *et al*. has suggested that either serum or glucose depletions increased mitochondrial fragmentation and induced mitochondrial tubular fission in MEFs, COS-7, HCT116, Atg5KO, and HEK293T cell lines [54]. Considering these, mitochondrial TRIP-Br1 may act as an oncoprotein by blocking mitophagy and ultimately aggravating tumorigenesis. The functional role of TRIP-Br1 in mitochondria needs to be investigated more. Our next project will be the study about the function of TRIP-Br1 in mitophagy. Quality control of mitochondria through manipulation of mitophagy could be an interesting project in cancer research. Our future studies also will aim to elucidate the interplay among autophagy, apoptosis, and necroptosis by studying the regulatory circuits governing them. In this process, it would be worthwhile to focus on mitochondria as the point of convergence of signaling pathways of autophagy, apoptosis, and necroptosis.

In addition to our valuable current studies, it should be noted that many researchers are using serum-starved conditions for several hours or days to synchronize cells or for drug treatment. However, because this practice causes significant changes in the gene expression of several genes such as TRIP-Br1, special care seems to be taken when serum starvation is used.

The ultimate goal is to unravel the optimal strategy for selectively killing cancer cells and developing an anticancer drug that causes no side effects in normal cells. In conclusion, our findings suggest that alleviating the positive effect of TRIP-Br1 oncoprotein on cancer cell survival could contribute to the development of anticancer drugs in more effective ways with other systemic therapies. Therefore, we suggest that TRIP-Br1 could be a good candidate, when combined with nutrient-starvation therapy, to efficiently kill cancer cells.

## MATERIALS AND METHODS

### Cell lines, cell culture, and other materials

Each cell line was cultured in one of the following media. Seven breast cancer cell lines (MCF7, MDA-MB-231, T47D, MDA-MB-453, Hs578D, BT549, and MDA-MB-435), two cervical cancer cell lines (SiHa and HeLa), and normal human and mouse cell lines (NIH3T3, HfCH8, and MEF) were cultured in Dulbecco's Modified Eagle's Medium (DMEM, WelGENE Inc., Korea) supplemented with 10% fetal bovine serum (FBS) (Gibco BRL, U.S.A) and 1% Antibiotic-Antimycotic solution (Gibco BRL, Cat#15240-062, U.S.A). Normal human MCF10A mammary epithelial cells were grown in DMEM/F12 medium (Invitrogen, Cat#11330-032, U.S.A) supplemented with 20 ng/ml of epidermal growth factor (EGF) (Sigma-Aldrich, Cat#E9644, U.S.A), 100 ng/ml of cholera toxin (Sigma-Aldrich, Cat#C-8052, U.S.A), 10 μg/ml of insulin (Sigma-Aldrich, Cat#I-9278, U.S.A), 0.5 mg/ml of hydrocortisone (Sigma-Aldrich, Cat#H-0888, U.S.A), 5% horse serum (Invitrogen, Cat#16050-122, Korea), and 1% Antibiotic-Antimycotic solution. All cells were cultured at 37°C in a humidified atmosphere composed of 95% air and 5% CO_2_. The SiHa cell line was obtained from the Korean Cell Line Bank (KCLB #30035); other cell lines were purchased from the American Type Culture Collection (ATCC). Materials used in this study were purchased from the following companies; Cobalt(II) chloride (CoCl_2_)(Sigma-Aldrich, Cat# STBC9672V, Korea), insulin-like growth factor 1(IGF-1)(Sigma-Aldrich, Cat#I3769, U.S.A), insulin (Sigma-Aldrich, Cat#I9278, U.S.A), EGF (Sigma-Aldrich, Cat#E9644, U.S.A), and LY294004 (Calbiochem, Cat#440202, U.S.A).

### Induction of glucose, amino acid, and serum starvation

Cells were placed in a complete medium or in a nutrient starved medium (free of glucose, amino acids, or serum) for the time periods. For the glucose-sufficient or glucose-deficient media, cells were cultured in 4.5 g/L of glucose containing DMEM (Gibco Invitrogen, Cat#LM001-05, Korea) or glucose-free DMEM (Gibco Invitrogen, Cat#11966-025, Korea) for 48 hours, during which 10% FBS was added to both media. For amino acid starvation, cells were first grown in DMEM supplemented with 10% FBS. At 80% confluence, the cells were washed with phosphate buffered saline (PBS) and incubated in Earle's Balanced Salt Solution (EBSS), amino acid-free medium (Gibco Invitrogen, Cat#14155-063, Korea) containing 5% bovine serum albumin (BSA), 0.1 mg/ml of MgCl_2_, 20 mM of HEPES, and 1% Antibiotic-Antimycotic solution for the lengths of time indicated. For serum-starvation conditions, cells were maintained in DMEM (Gibco Invitrogen, Cat#LM001-05, Korea) or DMEM/F12 (Biowest, Cat# L0092-500, France) without FBS or horse serum for the lengths of times indicated.

### Western blot analysis

Cells were centrifuged, washed in ice-cold PBS buffer, and then lysed in RIPA lysis buffer. The amount of protein was quantified using a protein assay kit (Bio-Rad, Korea). Each sample was subjected to SDS-PAGE and transferred to an Immobilon Transfer Membrane (Millipore, Cat#IPVH00010). The filter was incubated with each corresponding antibody, and immunodetection was carried out using the PowerOpti-ECL Western blotting detection reagent (Bio-Rad). The antibodies used in this study were purchased as follows: Bax (Santa Cruz, sc-20067), cyclophilin A (CypA)(Enzo Life Sciences, BML-SA296), HIF-1α (Cell Signaling, Cat#3716S), HMBG1 (Enzo Life Sciences, ALX-210-964), LC3 (Enzo Life Sciences, ALX-803-082), p62/SQSTM1(Cell Signaling, Cat#5114), RIP3 (Santa Cruz, sc-47368), TRIP-Br1 (Enzo Life Sciences, ALX-804-645), XIAP (Cell Signaling, Cat# 2042) and γ-tubulin (Santa Cruz, sc-7396).

### Silencing of TRIP-Br1 and XIAP genes

For transient knockdown of TRIP-Br1, the cells were transfected with TRIP-Br1 silencing siRNA using Lipofectamine 2000 (Invitrogen) in Opti-MEM medium, in which scrambled RNA (scRNA) was used as a nonsilencing control. After 5 hour incubation at 37°C, the transfection medium was replaced with DMEM with Antibiotic-Antimycotic. TRIP-Br1 silencing siRNA was purchased from Santa Cruz (Cat#sc-62988). In addition, TRIP-Br1 expression was also silenced with the use of GIPZ Lentiviral shRNA human SERTAD1 (Thermo Fisher Scientific Inc., Thermo Scientific™ GIPZ Lentiviral™ shRNA Library, V2LHS-64483), in which a GIPZ nonsilencing Lentiviral shRNA control vector was used as a nonsilencing control. The TRIP-Br1 silenced MCF7 stable cell line was selected with the use of 1 μg/ml of puromycin. For XIAP knock-down, the cells were transfected with XIAP silencing siRNA with the use of TurboFect Transfection Reagent (Thermo Scientific, Cat#R0531, Lithuania), in which scRNA acted as a nonsilencing control. After a 24 hour incubation in DMEM with 10% FBS and 1% Antibiotic-Antimycotic, the transfection medium was replaced with a fresh batch of the same medium. XIAP silencing siRNA was purchased from Cell Signaling Technologies (XIAP siRNA I, #6446, U.S.A).

### Analysis of autophagy, apoptosis, and necroptosis

Autophagy, apoptosis, and necroptosis were mainly analyzed by Western blotting with corresponding marker or regulatory proteins: p62 and LC3 for autophagy; Bax, XIAP, and PARP for apoptosis; HMGB1, CypA, and RIP3 for necroptosis. necroptosis was checked by measuring the levels of extracellular HMBG1 and CypA biomarker proteins that are released from necrotic and necroptotic cells to an extracellular location [29].

### Measurement of reactive oxygen species (ROS)

Cells were serum-starved for 24 or 48 hours and collected in 15 ml conical tubes and spun at 1,000 rpm for 2 minutes. Approximately ∼5 × 10^4^ cells in 50 μl of PBS were separated into 96 well plates, and 50 μl of 200 μM DCFH-DA (2′,7′-Dichlorodihydro fluorescein diacetate assay) was added with multichannel-pipettes (PIPETMAN, Gilson) into each well to achieve the 100 μM final concentration of DCFH-DA. ROS levels were detected by measuring the amount of fluorescence from 2′,7′-Dichlorodihydro fluorescein diacetate, in which fluorescence was measured at ex485/em535nm every 5 minutes for 30 minutes with a micro-plate reader.

### Mitochondrial fractionations

Mitochondrial fractionation was performed as previously described [23].

## SUPPLEMENTARY MATERIALS FIGURES



## Abbreviations

TRIP-Br1: Transcriptional regulator interacting with the PHD-bromodomain 1
ROS: Reactive Oxygen Species
XIAP: X-linked inhibitor of apoptosis protein
PCD: Programed cell death
