# Supplementary material for: TRIP-Br1 oncoprotein inhibits autophagy, apoptosis, and necroptosis under nutrient/serum-deprived condition
Source: Oncotarget. 2015 Aug 21;6(30):29060–75. doi: 10.18632/oncotarget.5072 (PMC4745711; doi:10.18632/oncotarget.5072)
Supplement: Supplementary file 1 [file oncotarget-06-29060-s001.pdf]

SUPPLEMENTARY FIGURES

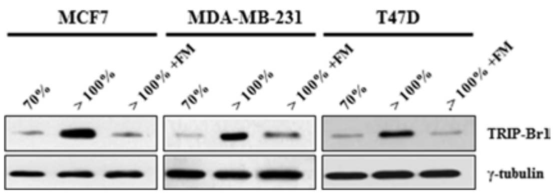

Supplementary Figure S1: Effect of overcrowded environment on the TRIP-Br1 expression level in MCF7, MDA-MB-231, and T47D breast cancer cells. Cells were cultured in complete media until they either reached approximately 70% confluence or were overcrowded, with high levels of cell confluence by prolonged culturing. Among them, one set of the overcrowded cells were used for further study, in which fresh media (FM) were added to them and cultured for 24 hours more. The cells were then collected and TRIP-Br1 expression levels were measured by means of Western blot analysis.

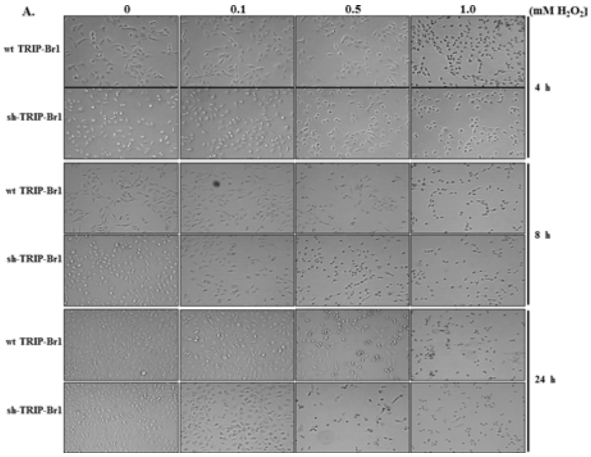

Supplementary Figure S2: Microscopic phenotype of TRIP-Br1 wild-type or knock-down MCF7 cells in response to H_2O_2 treatment. Cells were treated with 0.0, 0.1, 0.5, 1.0 mM of H_2O_2 for 4, 8, and 24 hours and then their phenotypes were photographed under the microscope.
